# Interpretable prediction models for widespread m6A RNA modification across cell lines and tissues

**DOI:** 10.1093/bioinformatics/btad709

**Published:** 2023-11-23

**Authors:** Ying Zhang, Zhikang Wang, Yiwen Zhang, Shanshan Li, Yuming Guo, Jiangning Song, Dong-Jun Yu

**Affiliations:** School of Computer Science and Engineering, Nanjing University of Science and Technology, Nanjing 210094, China; Biomedicine Discovery Institute and Department of Biochemistry and Molecular Biology, Monash University, Melbourne, VIC 3800, Australia; School of Public Health and Preventive Medicine, Monash University, Melbourne, VIC 3004, Australia; School of Public Health and Preventive Medicine, Monash University, Melbourne, VIC 3004, Australia; School of Public Health and Preventive Medicine, Monash University, Melbourne, VIC 3004, Australia; Biomedicine Discovery Institute and Department of Biochemistry and Molecular Biology, Monash University, Melbourne, VIC 3800, Australia; Monash Data Futures Institute, Monash University, Melbourne, VIC 3800, Australia; School of Computer Science and Engineering, Nanjing University of Science and Technology, Nanjing 210094, China

## Abstract

**Motivation:**

RNA N6-methyladenosine (m6A) in *Homo sapiens* plays vital roles in a variety of biological functions. Precise identification of m6A modifications is thus essential to elucidation of their biological functions and underlying molecular-level mechanisms. Currently available high-throughput single-nucleotide-resolution m6A modification data considerably accelerated the identification of RNA modification sites through the development of data-driven computational methods. Nevertheless, existing methods have limitations in terms of the coverage of single-nucleotide-resolution cell lines and have poor capability in model interpretations, thereby having limited applicability.

**Results:**

In this study, we present CLSM6A, comprising a set of deep learning-based models designed for predicting single-nucleotide-resolution m6A RNA modification sites across eight different cell lines and three tissues. Extensive benchmarking experiments are conducted on well-curated datasets and accordingly, CLSM6A achieves superior performance than current state-of-the-art methods. Furthermore, CLSM6A is capable of interpreting the prediction decision-making process by excavating critical motifs activated by filters and pinpointing highly concerned positions in both forward and backward propagations. CLSM6A exhibits better portability on similar cross-cell line/tissue datasets, reveals a strong association between highly activated motifs and high-impact motifs, and demonstrates complementary attributes of different interpretation strategies.

**Availability and implementation:**

The webserver is available at http://csbio.njust.edu.cn/bioinf/clsm6a. The datasets and code are available at https://github.com/zhangying-njust/CLSM6A/.

## 1 Introduction

The post-transcriptional modification (PTM) occurs in all living organisms and is evolutionarily conservative and functional (Sun *et al.* 2014). Currently, more than 170 types of PTMs have been identified in living organisms (Boccaletto *et al.* 2017, Roundtree *et al.* 2017), spanning multiple RNA types including mRNAs, tRNAs, rRNAs, lncRNAs, and snoRNAs (Jaffrey 2014, Gilbert *et al.* 2016). Among them, N6-methyladenosine (m6A), which was discovered in 1950 (Desrosiers *et al.* 1974), is the most typical and pervasive PTM. Specifically, it plays critical roles in various biological processes [e.g. mRNA metabolism (Wang *et al.* 2014), RNA splicing (Mendel *et al.* 2021), RNA stability and structure (Roost *et al.* 2015), DNA damage (Xiang *et al.* 2017), embryo development (Zhong *et al.* 2008), and cell apoptosis (Ping *et al.* 2014)]. Moreover, various human diseases are also closely related to m6A modifications (Meyer *et al.* 2012, Bansal *et al.* 2014, Cai *et al.* 2019, Esteve-Puig *et al.* 2020, Yu *et al.* 2021). Despite extensive research on m6A modification, the current knowledge is limited in understanding the regulatory mechanisms in different cell lines, the diverse functions of m6A modification, and the topological patterns within transcripts and across the genome. Therefore, the identification of m6A modification sites in the transcriptome is of great importance, which is essential to elucidate the biological functions and the underlying mechanisms.

Benefiting from the advancements in bioinformatics and next-generation sequencing (NGS) technologies, abundant high-throughput experimental data has been accumulated for identifying m6A RNA sites at a whole-genome scale. These experimental technologies can be categorized into two streams. The first is antibody-based methods, which are capable of identifying m6A-containing sequence fragments (low-resolution), including Methylated RNA Immunoprecipitation (MeRIP) (Meyer *et al.* 2012), m6A sequencing (m6A-seq) (Dominissini *et al.* 2012), and photo-crosslinking-assisted (PA-m6A-seq) (Chen *et al.* 2015). The second is high-resolution methods, which are able to identify m6A modification sites at a single-nucleotide level, such as m6A-crosslinking immunoprecipitation (CLIP) (Ke *et al.* 2015), individual-nucleotide-resolution cross-linking and immunoprecipitation (miCLIP) (Linder *et al.* 2015), DART-seq (Meyer 2019), MAZTER-Seq (Pandey and Pillai 2019), and m6A-REF-seq (Zhang *et al.*). Although the wet-lab experiments serve as the basis for revealing regulatory mechanisms of PTM, they are significantly limited by the time-consuming detection programs and expensive experimental materials.

The production of high-throughput biological experimental data and advances in artificial intelligence have piqued research interest in developing efficient and cost-effective computational methods for identifying m6A modification. Supplementary Table S1 comprehensively summarizes existing approaches for predicting m6A RNA in *Homo sapiens*. Machine learning-based methods such as SRAMP (Zhou *et al.* 2016), MethyRNA (Chen *et al.* 2017), iRNA-PseCol (Feng *et al.* 2017), RAM-NPPS (Xing *et al.* 2017), M6AMRFS (Qiang *et al.* 2018), HMpre (Zhao *et al.* 2018), WHISTLE (Chen *et al.* 2019), and iRNA-m6A (Dao *et al.* 2020), Convolutional Neural Network (CNN) based methods such as Gene2Vec (Zou *et al.* 2019), DeepM6ASeq (Zhang and Hamada 2018), DeepPromise (Chen *et al.* 2020), im6A-TS-CNN ([Bibr btad709-B23][Bibr btad709-B23]), TS-m6A-DL (Abbas *et al.* 2021), MultiRM (Song *et al.* 2021), and methods (Huang *et al.* 2018, Xiong *et al.* 2021) combining different classifiers are included. It is obvious that substantial efforts have been dedicated to the identification of m6A modification sites, leading to notable progress in this field. However, it has been reported that a subset of m6A modification is tissue-specific (Liu *et al.* 2020a; Zhang *et al.* 2019), whereas most computational tools ignore this critical insight. WHISTLE (Chen *et al.* 2019) particularly considers six specific cell lines in the benchmark datasets, however, it mixed these data together as the training set rather than regarding each cell line as an independent dataset. For prediction based on single-nucleotide resolution data, only four approaches [SRAMP (Zhou *et al.* 2016), iRNA-m6A (Dao *et al.* 2020), im6A-TS-CNN ([Bibr btad709-B23][Bibr btad709-B23]), and TS-m6A-DL (Abbas *et al.* 2021)] take cell line/tissue condition into account clearly and distinguish the identification of cell line/tissue specific m6A modification. SRAMP (Zhou *et al.* 2016), which is constructed by three random forest classifiers, explores extensive cell lines and tissues such as HEK293 cell, CD8+ T cell, A549 cell, brain, and liver. However, it fails to achieve a satisfying accuracy. iRNA-m6A (Dao *et al.* 2020), im6A-TS-CNN (Liu *et al*. 2020b), and TS-m6A-DL (Abbas *et al.* 2021) are restricted to specific tissue types (brain, kidney, and liver) and are weak in interpreting the prediction process both in individual cell lines and across different cell lines, thus impeding the understanding of the underlying mechanisms. To this end, it is crucial to develop single-nucleotide resolution approaches that encompass a broader range of cell lines and tissues and derive explanatory biological information from the well-trained models.

In this study, we present a CNN-based neural network to identify the **C**ell **L**ine **S**pecific **m6A** sites at single-nucleotide resolution, termed CLSM6A. CLSM6A covers eight cell lines (A549, CD8T, HCT116, HEK293, HEK293T, HeLa, HepG2, MOLM13) and three tissues (brain, kidney, liver), which are more comprehensive than existing single-nucleotide resolution-based cell line/tissue-specific approaches for identifying m6A modification sites in *H. sapiens*. Moreover, we interpret the prediction process by excavating critical motifs and patterns and conducting forward and backward propagations, in which process we found that nucleotides at proximal positions surrounding the modification sites contributed more to the classification.

## 2 Materials and methods

### 2.1 Benchmark datasets

**Figure 1.**
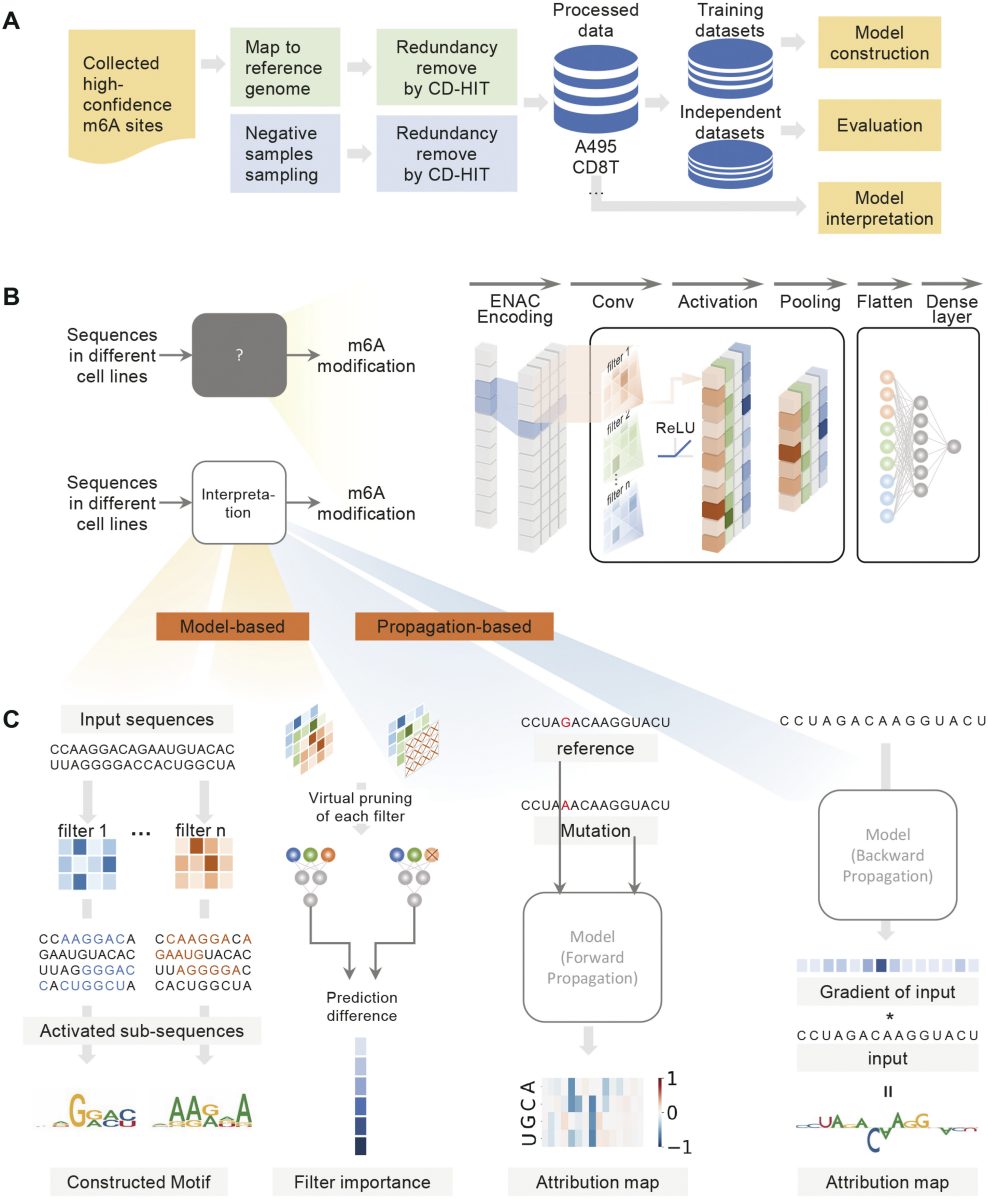
Overview of the proposed CLSM6A framework. (A) Collection and processing of m6A RNA modification data. (B) Structure of the proposed CLSM6A in this study. (C) Model-based and propagation-based interpretation.

High-quality datasets are crucial for the construction of reliable predictors. Here, the cell line specific m6A RNA modification sites in *H. sapiens* were obtained from m6A-Atlas (Tang *et al.* 2021). It is a high-confidence knowledgebase of reliable m6A sites that have been identified by base-resolution technologies, which covers various tissues and cell lines. The framework of data processing is illustrated in Fig. 1A. Firstly, modification sites from the same cell line/tissue were merged and mapped to the reference genomes (http://www.ensembl.org). After that, the sequences filtered, only retaining those under DRACH motifs (where D = “A”, “G” or “U”; R = “A” or “G”; and H = “A”, “C” or “U”) (Dominissini *et al.* 2012, Meyer *et al.* 2012, Chen *et al.* 2015, Linder *et al.* 2015). To construct the negative datasets, we implemented two sampling procedures. In the first step, the following rules were adopted: (i) all m6A sites were excluded during sampling of negative samples, (ii) each negative modification site was at least 200 nt away from any positive modification site to avoid overlapping with positive sequences, and (iii) the “DRACH” motif was used to filter the data. In the second step, we sampled the result of the first step to obtain a balanced benchmark dataset for each cell line/tissue. All the sequences were extracted based on the mature mRNA mode.

For each m6A or non-m6A site, a 201 nt sequence was extracted with adenine in the center. Then the CD-HIT program (Fu *et al.* 2012) was conducted on each line/tissue to overcome redundancy and reduce the homology bias to remove similar sequences with the threshold of 80%. Finally, we obtained 11 datasets from different cell lines and tissues in *H. sapiens*. The detailed information of these benchmark datasets are provided in Table 1. After this procedure, each dataset was randomly divided into training and independent test datasets with a ratio of 9:1. The training datasets were used for model construction and the independent test datasets were used for evaluation.

**Table 1.** Summary of cell line/tissue specific benchmark datasets curated in this study.

Cell line/tissue	No. of positive samples	Technique	GSE
A549	9344	m6A-seq with improved protocol	GSE54365
		m6A-CLIP-seq	GSE71154
Brain	3919	m6A-REF-seq	GSE125240
CD8T	12 352	m6A-CLIP-seq	GSE71154
HCT116	4069	miCLIP	GSE128699
HEK293	8221	miCLIP	GSE63753
HEK293T	44 317	miCLIP	GSE73405
		miCLIP	GSE122948
		MAZTER-seq	GSE122961
		m6A-REF-seq	GSE125240
HeLa	14 318	PA-m6A-seq	GSE54921
		m6A-CLIP-seq	GSE86336
HepG2	7062	miCLIP	GSE73405
		miCLIP	GSE121942
Kidney	3402	m6A-REF-seq	GSE125240
Liver	1574	m6A-REF-seq	GSE125240
MOLM13	15 261	miCLIP	GSE98623

### 2.2 The CLSM6A algorithm

The structure of the proposed CLSM6A is illustrated in Fig. 1B. The models for specific cell lines are similar in components, including the encoding block, the convolution block, and the classification block. The model is described sequentially from the input sequence to predictions, where the output of one layer is fed into the next layer in order.

1. Input and encoding block:
  The model takes RNA sequences of length L(L=2n+1) as inputs. To capture the nucleotide composition around RNA modification sites, the nucleotides in the sequence are represented using ENAC encoding with a sequence length of 2 and a sliding-window size of 1. Thus, each sequence is transformed to a 2D matrix size of (L−1)×4 after the encoding.
2. Convolution block:
  The encoding sequences are fed into several convolution blocks. Each convolution block consists of a convolutional layer (ConvNet), a rectified linear activation (ReLU) unit, a max pooling layer, and dropout regularization.
3. Classification block:
  The tensor obtained from the last convolution layer is concatenated and then fed into the fully connected layer. Eventually, the output is fed into the output layer with a single neuron followed by a sigmoid function to predict the sequence label.

To avoid overfitting, dropout was utilized at the end of all the convolutional blocks and the fully connected layer (Srivastava *et al.* 2014). Binary cross-entropy was used as the loss function, which calculates the difference between the predicted label distribution and the true observed label. All parameters were optimized using Adam on the loss function.

A total of 11 classifiers were introduced for the cell lines and tissues, with all of them constructed by the above blocks. Considering that the number of positive samples varies among different cell lines and tissues, we accordingly adjust the sizes of convolution blocks and neurons in the fully connected layer of the classification blocks to align the model complexity with the data volume. The detailed hyperparameters of each model were summarized in Supplementary Table S2. For each cell line or tissue, 5-fold cross-validation was utilized to train and optimize the model on the training data to determine the optimal model structure and hyperparameters.

The models were implemented in PyTorch (version 1.7.1) and were trained on a single NVIDIA RTX 3090 GPU. The final hyperparameters are summarized in Supplementary Table S3. Model performance was evaluated on the testing datasets.

### 2.3 Model-based interpretation

Interpreting the decision-making process is critical for understanding the underlying mechanisms of m6A modification. Therefore, the model-based interpretation was utilized, as illustrated in the left panel of Fig. 1C by analyzing the extracted motifs. Specifically, the first convolutional layer was analyzed to extract motifs, where the filters provide a position-weight matrix (PWM). Following the strategy (Trabelsi *et al.* 2019), we searched for the subsequences in the testing datasets which activate a given filter above a chosen threshold (half of the filter’s maximum value). The set of activated subsequences was used to a construct the PWM, and the nucleotide frequencies are counted to form a position frequency matrix (PFM). Then, sequence logos were constructed by the R package ggseqlogo.

We defined “activated amount” and “impact score” to measure the contribution of each filter. The activated amount refers to the number of activated subsequences above a chosen threshold (Trabelsi *et al.* 2019) in the testing datasets for each filter. The average number of activated subsequences was used to quantify the activated degree. The impact score represents the impact of filters on the model’s predictions by the virtual pruning strategy. Specifically, we nullified each filter in turn by setting its parameters to zero and measured its impact on the model’s predictions. Generally, nullifying an important filter would significantly alter the model’s predictions, while nullifying a filter with small importance would have little effect, instead.

### 2.4 Propagation-based interpretation

In addition, we adopt propagation-based interpretation, as illustrated in the right panel of Fig. 1C. This is considered as a post hoc interpretation strategy, which includes forward propagation and backward propagation.

- Forward propagation of influence
  The *in silico* mutagenesis (ISM) (Zhou and Troyanskaya 2015) strategy, which is similar to the popular perturbation strategies in computer vision (Samek *et al.* 2016) was employed to distinguish key areas for making predictions. Specifically, at each position of a given input RNA sequence $X=\left[x_{1},x_{2},\ldots ,x_{i},\ldots ,x_{l}\right]$, where $x_{i}$ is the *i*th base of the sequence and l is the length of the sequence, $x_{i}$ was replaced by three alternative nucleotides respectively, generating a new sequence each time. CLSM6M made prediction for both the original sequence and the alternative sequence, and the difference between the two predictions was termed as attribution score. For each original sequence, all the attribution scores resulted in an attribution map, which is a 4×l matrix.
- Backward propagation of influence
  Different from ISM, back-propagation calculated the derivative of the model at a given input sequence to evaluate the impact of each input elements. The results were adjusted to provide an attribution map size of 4×l for each sequence.

### 2.5 Performance measurement

To evaluate the performance of CLSM6A, several assessment metrics were employed, namely sensitivity (Sen), specificity (Spe), accuracy (Acc), and Matthew’s correlation coefficient (MCC), which are defined as follows:


(1) $$\left\{ \begin{array}{l} Sen=\frac{TP}{TP+FN}\\ Spe=\frac{TN}{TN+FP}\\ Acc=\frac{TP+TN}{TP+FP+TN+FN}\\ MCC=\frac{TP\times TN-FP\times FN}{\sqrt{\left(TP+FP\right)\times \left(TN+FN\right)\times \left(TP+FN\right)\times \left(TN+FP\right)}} \end{array}\right.$$

where TP, FP, TN, and FN represent the number of true positive, false positive, true negative, and false negative, respectively. Both Acc and MCC are used to measure the overall performance of the models, with MCC values ranging from −1 to 1 and Acc values ranging from 0 to 1. The receiver-operating characteristic (ROC) curves and precision–recall curves (PRC) were additionally plotted to comprehensively evaluate the performance of the models. The threshold-independent AUC and AP values were calculated based on the ROC curves and PRC, respectively.

## 3 Results and discussion

### 3.1 CLSM6A accurately predicts m6A sites in different cell lines and tissues

To assess the performance of the proposed CLSM6A, we evaluated it on eight cell lines and three tissues benchmark datasets in *H. sapiens*. Detailed information on these datasets is provided in the “Benchmark datasets” section. We particularly compared CLSM6A with two other deep-learning-based predictors [im6A-TS-CNN ([Bibr btad709-B23][Bibr btad709-B23]) and TS-m6A-DL (Abbas *et al.* 2021)]. Both of them are CNN-based predictors that utilize one-hot encoding scheme. Considering that the compared methods only cover three tissues in *H. sapiens*, we rebuilt the models according to the descriptions in the respective papers. The structures, optimizers, and early stopping strategy were maintained, and the well-trained models were used for the evaluation. All the methods used the same training and datasets as CLSM6A to ensure a fair performance comparison. Besides, we trained im6A-TS-CNN and TS-m6A-DL in an additional mode (sequences with a length of 41 nt), as the originally published models were trained on sequences of such length.

**Figure 2.**
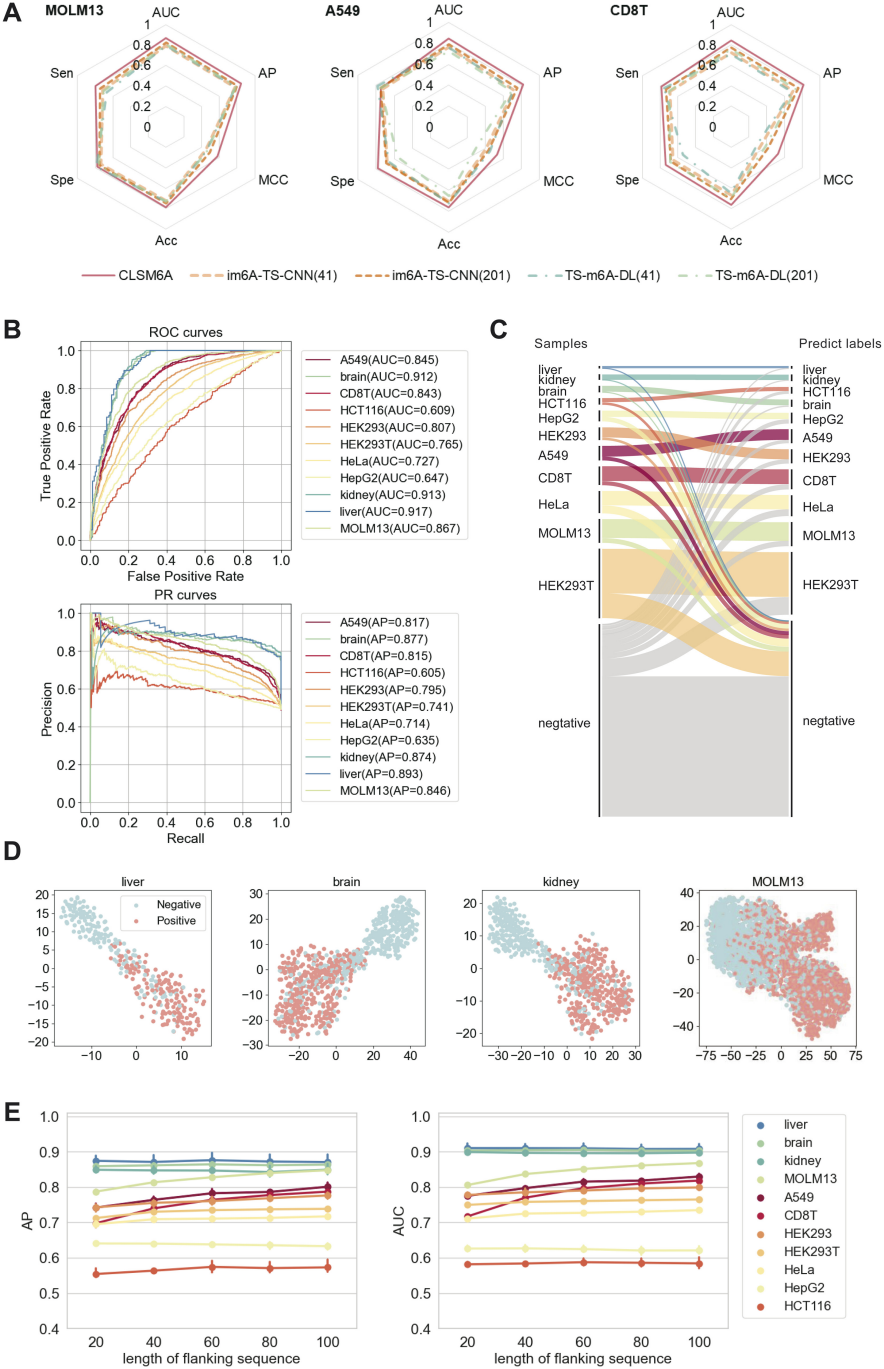
CLSM6A enables the single-nucleotide-resolution prediction of m6A sites. (A) Prediction performance on the three tissue testing sets in terms of area under the receiver-operating characteristic curve (AUC), the area under the precision–recall curve (AP), Matthew’s correlation coefficient (MCC), accuracy (Acc), specificity (Spe), and sensitivity (Sen). Considered were im6A-TS-CNN and TS-m6A-DL trained on the sequence length of 41 (the length of original model) and 201 (the length same as CLSM6A). (B) ROC curves and PR curves of CLSM6A in cell lines and tissues. (C) A Sankey diagram of the number of samples and the predicted results in the testing dataset. (D) The feature space distribution visualization in four cell lines/tissues. (E) Performance of CLSM6A under different length of flanking sequence.

Firstly, we varied the length of the flanking sequences from 20 nt to 100 nt at the step of 20 to find the suitable sequence length (experimental details in Supplementary Text S1). As shown in Fig. 2E, except for MOLM13, A549, CD8T, and HEK293, the AP and AUC values of other cell lines were stable with varied input sequence lengths. While regarding the four cell lines, the AP and AUC values increased and then reached a plateau with the increasing length of the flanking sequences. In this context, a consistent 100 nt flanking sequence length (201 nt of sequence length) was adopted on the benchmark datasets. The original im6A-TS-CNN and TS-m6A-DL were proposed and trained on sequences with the length of 41 nt. To ensure a fair and explicit comparison, the models were retrained to match CLSM6A’s sequence length, with results from their original sequence length further included.

Figure 2B, illustrates the performance of CLSM6A in terms of ROC curves and PR curves across tissues and cells. Specifically, the AUC and AP values on three tissues reach over 0.912 and 0.874, respectively. AUC and AP values on four cell lines reach over 0.8 and the AUC and AP values of the rest cell lines varies in 0.609–0.727 and 0.605–0.714, respectively. Figure 2C, is a Sankey diagram to intuitively visualize the number of samples in each cell line and the flow of quantity, ground truth to the predicted label. It is evident that CLSM6A successfully classifies most samples of each cell line/tissue. The performance of compared methods on the independent datasets are provided in Fig. 2A, Supplementary Fig. S1, and Supplementary Table S4. As shown in Supplementary Table S4, the proposed CLSM6A achieved the best average performance across all the evaluation metrics (AUC of 0.8040 and AP of 0.7811), reaching the highest AUC values in 10 from the 11 cell lines and tissues. Specifically, the AUC increases by about 4.27%–6.81% in A549, CD8T and MOLM13, demonstrating the significant superiority of CLSM6A.

Besides, to exhibit the feature engineering capability of CLSM6A, we visualized the positive and negative samples in the testing datasets using t-SNE (Van der Maaten and Hinton 2008). As shown in Fig. 2D and Supplementary Fig. S2, each point represents the feature of the last layer of the model, with the sample categories differently colored. The proposed CLSM6A separates methylated and non-methylated sites well. Specifically, features from a more accurate cell line specific model are more discriminative across different cell lines.

### 3.2 Learned motifs significantly matched with known motifs

**Figure 3.**
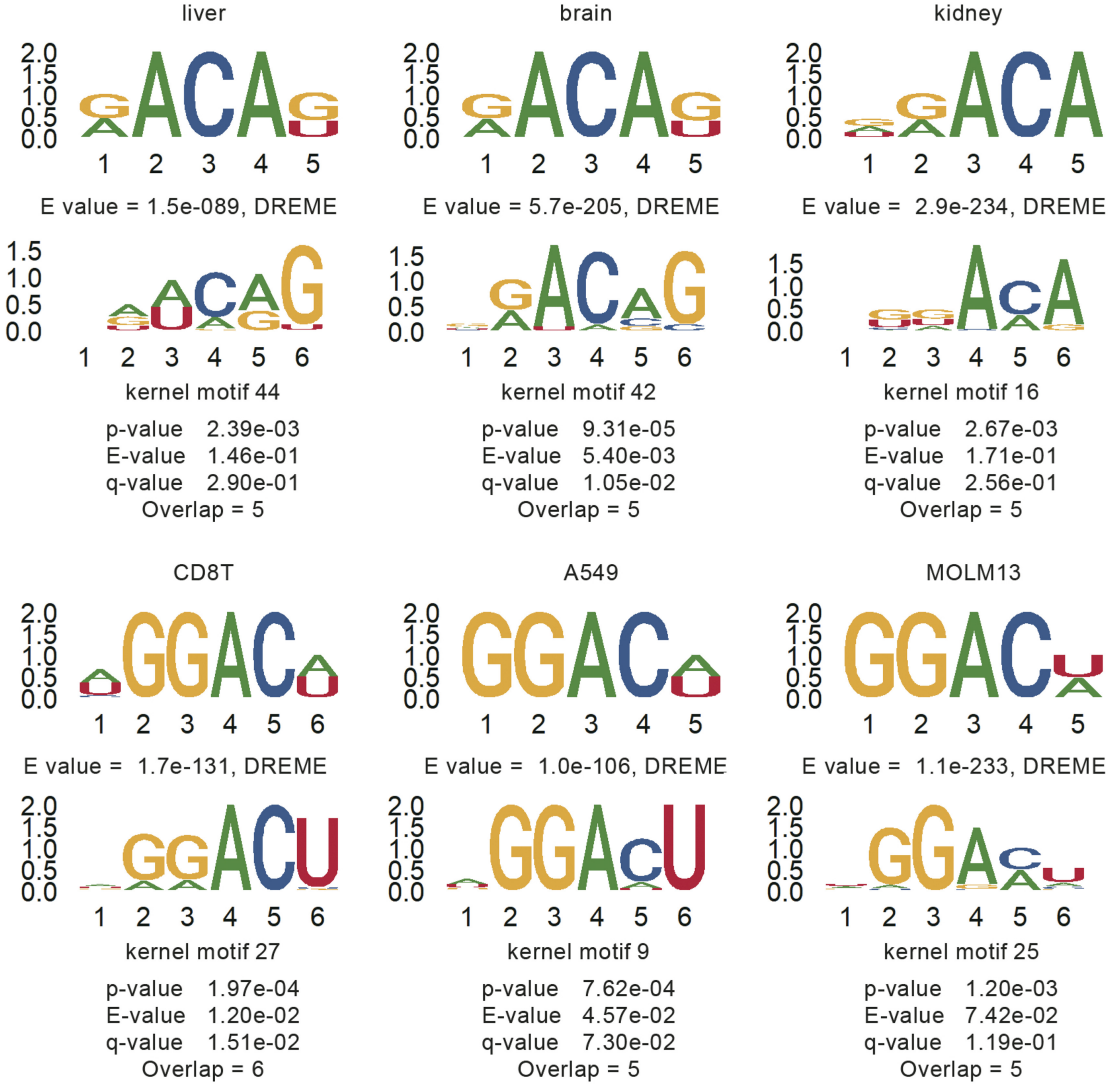
Characteristic motifs identified from conventional motif discovery tool DREME can be detected by the first convolutional layer of CLSM6A. For each aligned result, the upper panel is the motif (with the smallest E-value in each cell line) identified by DREME. With the E-values displayed below. The bottom panel is the motif detected by CLSM6A, with the kernel motif number, *P*-value and number of overlaps provided, and *P*-value was calculated using TOMTOM by utilizing a null model containing CLSM6A’s motif columns from the top column in the set of DREME motifs.

Identifying the learned patterns and motifs from the sequence is critical for model prediction interpretation. To this end, we employed the conventional motif finding method DREME (Bailey 2011) (Version 5.5.1) to unveil sequence patterns and adopted motif comparison tool TOMTOM (Gupta *et al.* 2007) (Version 5.5.1) for motif alignment and quantifying the similarity between the reported motifs by DREME and the extracted motifs by CLSM6A. For the well-trained models of the eight cell lines and three tissues, each of them has 64 filters in the first convolutional layer. Specifically, the motifs from the first convolutional layer of CLSM6A were extracted according to the process in “Propagation-based interpretation.” Regarding the positive samples in each cell line, we used DREME to search motif sites on given strand only under the threshold of E-value = 0.05. 11–49 motifs were reported for these cell lines. For each cell line, the first de novo motif with the lowest E-value was submitted into TOMTOM to find similar identified motifs by CLSM6A. *P*-value, E-value, q-value, and the number of overlaps were reported (Fig. 3 and Supplementary Fig. S3). For all the cell lines, CLSM6A is able to detect informative motifs that match well with the motifs unveiled from DREME with significant *P*-values (<0.05). Based on the above discussion, it can be inferred that the models specific to each cell line were able to capture significant sequence information and the findings of the model can assist the excavation of potential motifs.

### 3.3 CLSM6A reveals the relationship across different cell lines

**Figure 4.**
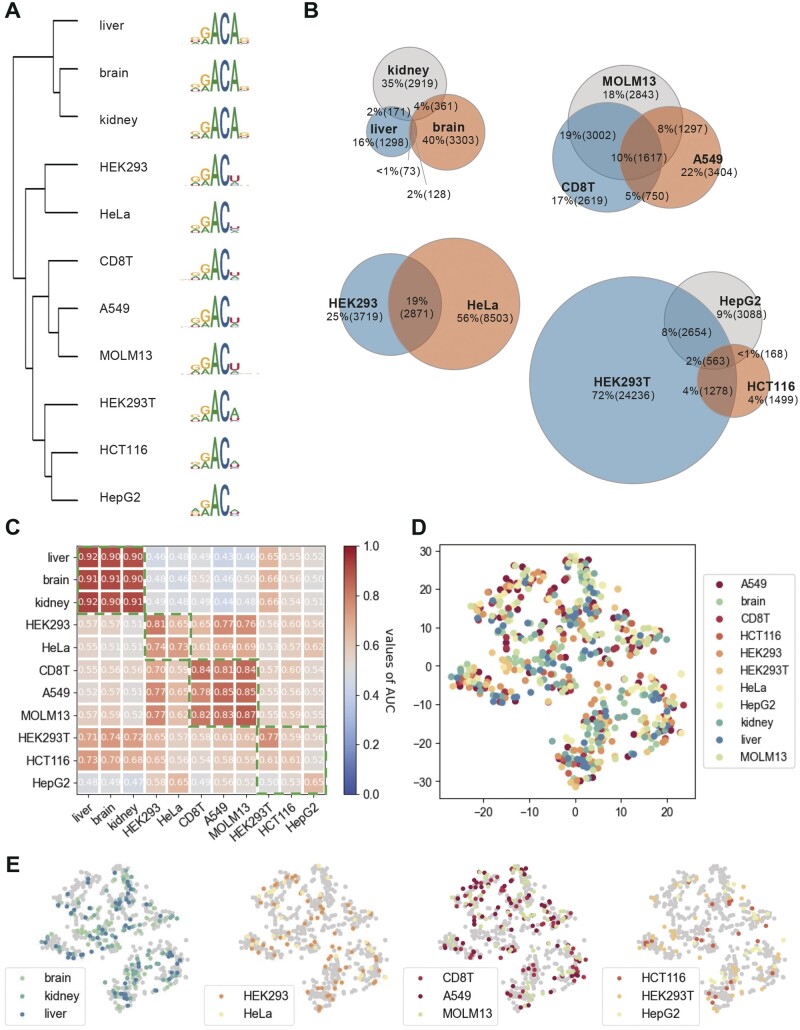
Illustration of the relationships between datasets and validation across lines and tissues. (A) Frequency-based clustermap of different cell lines and tissues, and the sequence motifs plotted by SeqLogo. (B) Venn diagram of modifications sharing the same exon. (C) Heatmap of cross-species validation with the used datasets on the *x*-axis and the models on the *y*-axis; a cell line/tissue specific model (in columns) was well-trained on its own training data and validated on the independent data on the cell line/tissue specific in rows. (D, E) The feature space distribution of activated motifs in cell lines/tissues colored by cell type.

Since m6A modifications are implicated in diverse biological processes that vary across different cell lines and tissues, they exhibit potential tissue specificity (Liu *et al.* 2020a). To explore the correlations across different cell lines and tissues, we clustered the dataset based on similarity, analyzed the intersection within each cluster, and conducted cross-cell line/tissue validation. Specifically, we utilized the positive samples of three tissues and eight cell lines and performed clustering of the similar datasets based on their frequency, following motif generation by SeqLogo. As illustrated in Fig. 4A, motif discrepancy among different cell lines and tissues can be observed. The samples were categorized into four groups on the cluster map. Within each group, the sequences exhibited similar motifs, indicating a degree of similarity in their underlying biological processes. For example, samples in liver, brain and kidney displayed highly comparable sequence motifs. The nucleotide distribution surrounding m6A and non-m6A sites, as revealed by Two Sample Logos, was also remarkably similar within groups (Supplementary Fig. S4). However, some m6A modification sites may only occur in specific cell lines/tissues and some may exhibit a propensity for cautious reporting in multiple lines and tissues. Therefore, we presented the modification intersection of each group in Fig. 4B. Specifically, we listed the exons associated with each modification, and considered modifications that shared the same exon as intersected. In the MOLM13, CD8T, A549, HepG2, and HCT116 cell lines, we found that more than half of the modification sites were shared among other cell lines/tissues. This suggests that there are both high correlations and notable differences between cell line and tissue data.

Our primary interest lies in the outcomes of cross-line/tissue validation based on the association among different cell lines/tissues. To achieve this goal, we utilized the 11 tissue/cell line-specific well-trained models to conduct cross-evaluation. The AUC values of cross-line/tissue validation are presented in the heatmap in Fig. 4C, in which the *x*-axis of the panels represents the dataset and the *y*-axis denotes the predictors. Warmer colors (the larger values) indicate a higher degree of transferability and generalization. The values of cell lines/tissues from the same group were marked by green dashed boxes. In terms of the first three groups, the within-group transferability demonstrates satisfactory performance and effectiveness, with AUC values ranging in 0.90–0.92, 0.65–0.81, and 0.78–0.87, respectively. This suggests that the models within these groups can identify potential m6A sites in group models. For validation in the fourth group, the reported AUC values were below 0.77 and were not significantly distinguished from cell lines/tissues outside the group. In conclusion, our cross-line/tissue validation results indicate that the model performance and the similarity between data are critical factors in model transferability across different cell lines and tissues. These findings provide valuable insights into the biological processes in which m6A modifications may be involved and suggest potential avenues for future research.

In the previous section, we demonstrated that CLSM6A was capable of capturing meaningful subsequences from the input data. Nevertheless, the relationship of captured motifs between cell lines is unclear. Therefore, we investigate the relationship between the captured motifs across cell lines. Specifically, we visualized the distribution of motifs by mapping the position frequency matrix (PFM) of each motif to the 2D space using t-SNE. Figure 4D exhibits the distribution of motifs of all the cell lines and tissues. Figure 4E presents a clearer exhibition of the distribution in each cluster group. Generally, the motifs were mixed together and exhibited heterogeneous clustering across different cell lines. Furthermore, parts of the points were overlapped or extremely close to each other, implying that the cell line specific models extract general motifs while similar motifs may be reported by different cell lines.

### 3.4 Virtual pruning uncovers the relationship between highly activated motifs and high-impact motifs

**Figure 5.**
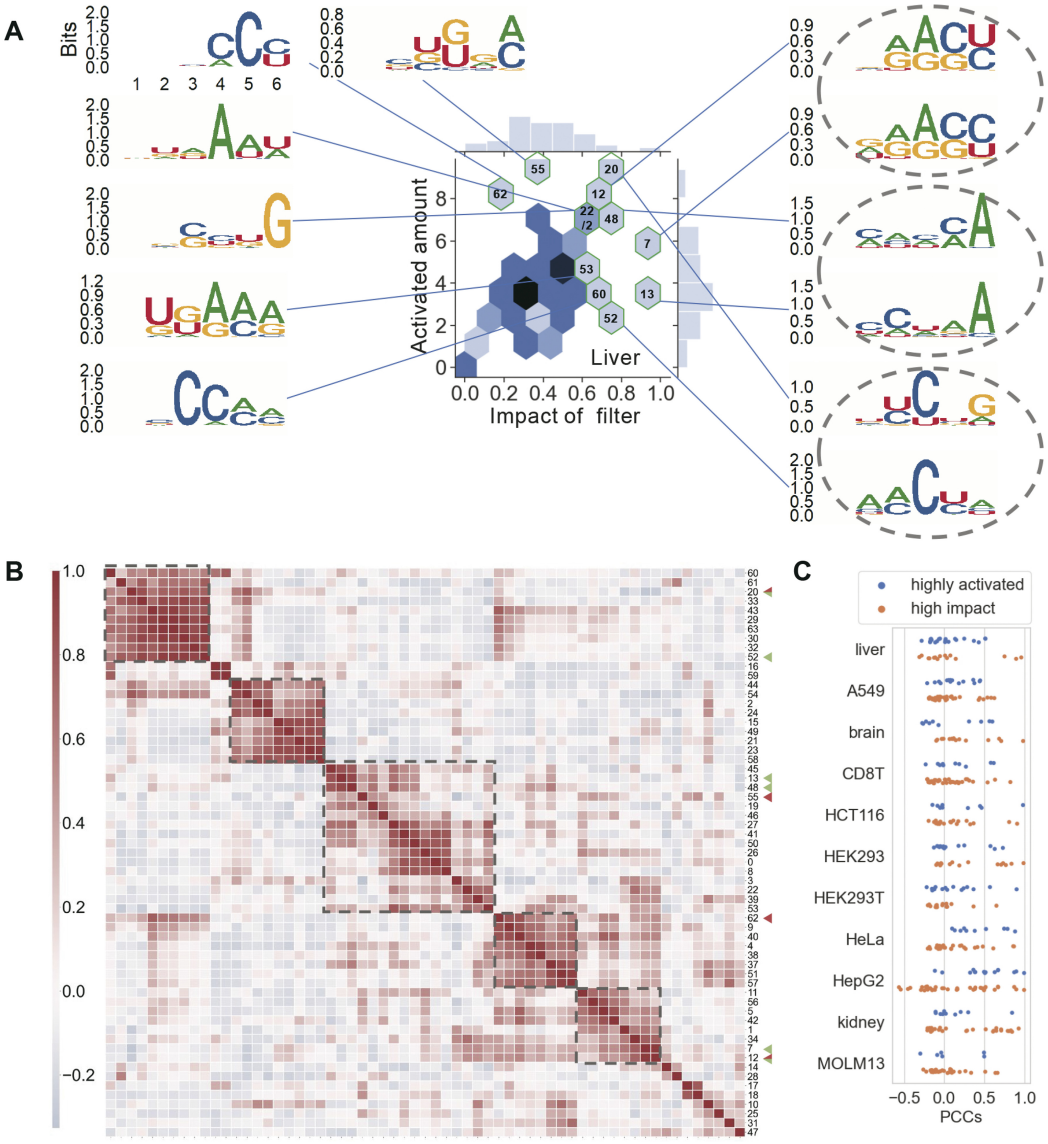
Analysis of motifs learned by CLSM6A. (A) Visualization of the motif distribution in the liver cell line, with the impact of filter on the *x*-axis and the average activated amount sequences on the *y*-axis. Motif filters with high impact or high activated amounts are displayed. (B) Correlation among motifs. Pairwise PCCs between motifs within the same cluster exhibit high values. The highly activated and high-impact motifs are marked in red and green, respectively. (C) Pairwise PCCs between top highly activated motifs and important motifs in 11 cell lines.

In this subsection, we aim to explore the relationship between the attentive motifs learned by CLSM6A. To accomplish this goal, we analyzed the important motifs that were highly activated through virtual pruning (Methods). Hereby, we utilized the impact score to estimate the filter effect by considering its contribution to m6A RNA modification, which is visualized in Fig. 5A and Supplementary Figs S5–S14, with the impact of filter on the *x*-axis and the average activated amount sequences on the y-axis. The distribution of impact scores and activated amounts of filters was observed to be either near-Gaussian or right-skewed. This implies that only a few motifs with larger activated amounts or higher impact are of greater importance. To measure the similarity of the extract motifs in each cell line, we computed pairwise Pearson correlation coefficients (PCCs) between the motifs’ position frequency matrix and then clustered the motifs, and marked motifs with large activated amounts in red and those with high impacts in green in Fig. 5B. It is shown that motifs can be clustered into several groups. The red markers tended to occur in different groups while the green markers are more likely to co-occur in the same cluster. This reveals that the filters from one model, especially those activating more sequences, tend to be different from each other, which enables the model to extensively learn a variety of potential features of the input sequences. For filter impact, the nullification of similar filters results in similar global alterations in the model’s predictions. Therefore, filters with high impact scores were more likely to be similar. In Fig. 5C, the PCCs between top high impact motifs and top highly activated motifs in 11 cell lines are calculated and reported. The observation indicates that motifs with a high impact tend to have higher PCCs than motifs that are highly activated.

### 3.5 Mathematical propagation of influence reveals different information from that of model-based interpretation

In this subsection, we simultaneously utilized the model-based interpretation and propagation-based interpretation to explain the attentive subsequences of the trained model. For the model-based interpretation, the activated subsequences were mapped onto the input sequences and the activated nucleotides (A/G/C/U) were counted separately at each position. If a subsequence was activated by two or more filters, the nucleotide count at the position was accumulated. As a result, a matrix size of 4×l was recorded for each sequence. For the propagation-based interpretation, the models’ attribution maps were analyzed using both forward and backward propagation. For the three strategies, we averaged the attribution maps of all the samples in the testing set and then convert the matrix to a vector to reveal a global understanding of concerned positions.

**Figure 6.**
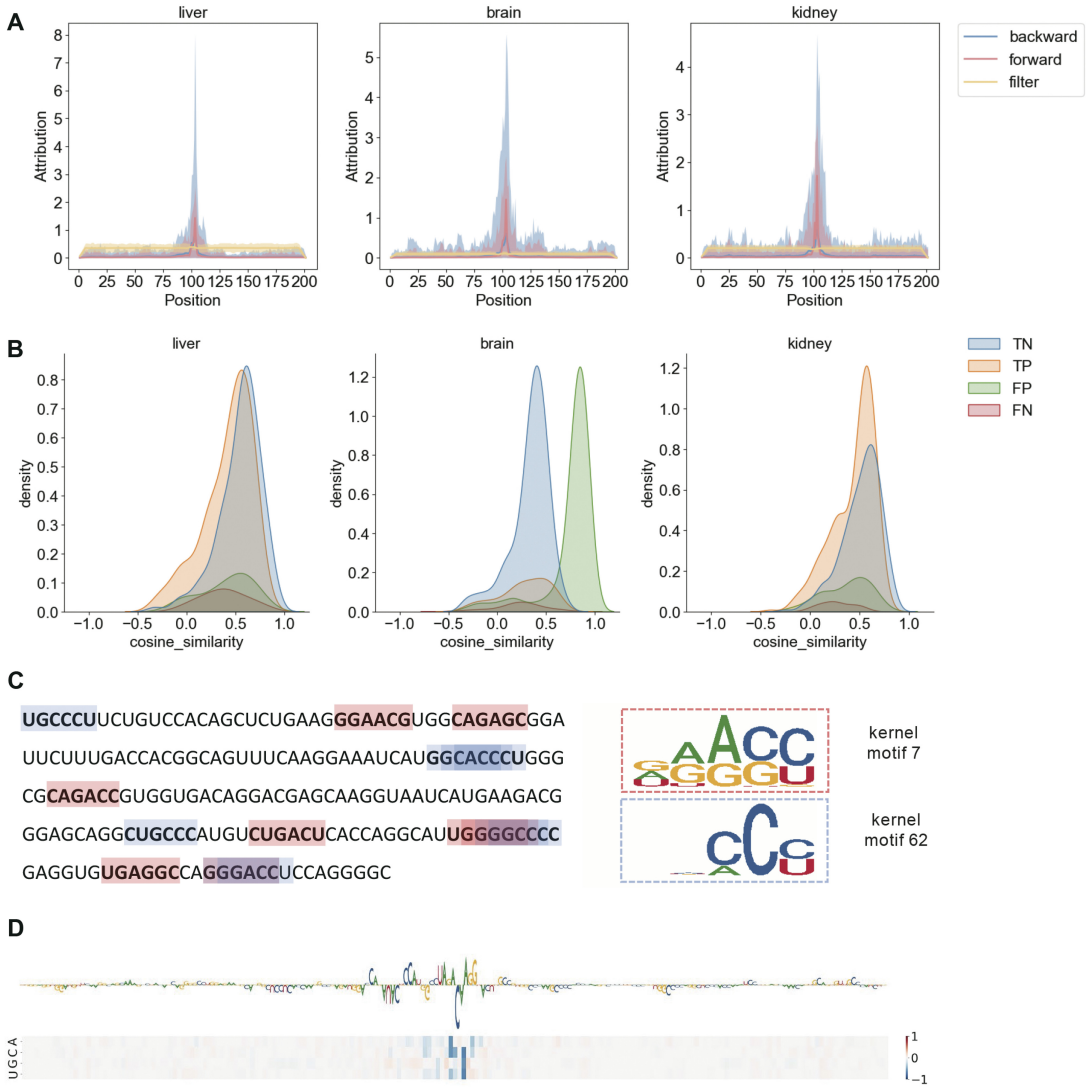
Model-based interpretation and the propagation-based interpretation on the tissue of liver brain and kidney. (A) Global exhibition of positions the model focuses on via three strategies, with solid lines recording the averaged values and the light background ranging from the smallest value to the largest value in each position. (B) Local (single input example) attribution similarity by the two propagation-based interpretation methods. (C) Local exhibition (single input example) of activated subsequences by different filters. (D) Exhibition (single input example) of attribution vectors reported by propagation-based interpretations.

As shown in Fig. 6A and Supplementary Fig. S15, in all cell lines and tissues, the global accumulated concerned positions of filters show distinct areas of interest compared to the two mathematical propagation-based strategies. Specifically, the filters’ globally accumulated concerned positions are evenly distributed over the entire sequence. For a clear exhibition, we randomly selected one sequence from the liver dataset and annotated the activated subsequences by two randomly selected filters. As shown in Fig. 6C, the positions of the subsequence are not centralized, which means CLSM6A has a global receptive field with motif-specific attention capability. Nevertheless, as shown in Fig. 6D, the two mathematical propagation-based strategies show different concerned areas from that of filters. Both the forward and backward propagations focus on emphasizing the middle of sequences, resulting in peak activations in the central area. Only if high values exist outside of the central, the central region will exhibit lower peaks.

Although forward propagation and backward propagations exhibit similar global attribution, the similarity for a specific sequence is still worth to be evaluated. Hereby, we compared the attribution vectors of each sample generated by the two strategies to assess their similarity and found that they differ from each other (average cosine similarity smaller than 0.5) (Fig. 6B and Supplementary Fig. S16). Model-based and propagation-based interpretations reveal the applicability of the respective. Concerned positions by filters, considered as model-based interpretation, are adept at learning for the specific subsequences, and this is consistent with the scanning characteristics of the filters. Propagation-based interpretations, considered as post hoc interpretation strategies, do not necessitate the utilization or analysis of specific modules and solely operate on a trained model, with quantifying the feature importance. Given the complementary nature of these two propagation-based strategies, both are encouraged to be used when researchers interpret the model prediction.

## 4 Conclusion

In this paper, we present CLSM6A, a deep learning-based pipeline for predicting m6A RNA modification sites at single-nucleotide resolution in a series of cell lines and tissues. Benchmarking experiments demonstrate the superiority of CLSM6A compared with other state-of-the-art methods. Extensive model interpretation and cross-cell lines/tissues analysis demonstrate the scalability and usability of CLSM6A. Specifically, motifs excavated by our proposed neural network are mostly scientifically certified by conventional motif-finding methods. Moreover, based on the cross-cell line/tissue validation, we found that data similarity is critical in model transferability.

All the motifs activated by filters in our model matched the motifs unveiled by conventional motif-finding methods, which significantly highlights the model's strong learning capability. Through the cross-cell line/tissue validation, we observed better portability existing in similar data. By analyzing the motifs extracted by the filters, we found that similar motifs may be reported by different cell lines. In a specific cell line, it was observed that motifs with higher activation tend to be more distinguished from each other, while those with higher impact scores are more likely to be similar. Moreover, we conducted model-based and propagation-based interpretations, which reveal different concerned positions, such complementary attributes encouraging the researchers to utilize both of them in interpreting the models.

Overall, our pipeline show promise as a useful tool for predicting m6A RNA modifications in cell lines and tissues. The strategies and techniques used in this study can be applied to similar tasks and the insights gained from analyzing the learned motifs and their relationships across different cell lines and tissues can provide valuable information for understanding the underlying mechanisms of m6A modifications.

## Supplementary Material

btad709_Supplementary_Data
