# Supplementary material for: Interpretable prediction models for widespread m6A RNA modification across cell lines and tissues
Source: Bioinformatics. 2023 Nov 23;39(12):btad709. doi: 10.1093/bioinformatics/btad709 (PMC10697738; doi:10.1093/bioinformatics/btad709)
Supplement: btad709_Supplementary_Data [file btad709_supplementary_data.doc]

**Interpretable prediction models for widespread m6A RNA modification across cell lines and tissues**

Ying Zhang^1^, Zhikang Wang^2^, Yiwen Zhang^3^, Shanshan Li^3^, Yuming Guo^3^, Jiangning Song^2,4^,*, and Dong-Jun Yu^1^,*

^1^School of Computer Science and Engineering, Nanjing University of Science and Technology, 200 Xiaolingwei, Nanjing, 210094, China;

^2^Biomedicine Discovery Institute and Department of Biochemistry and Molecular Biology, Monash University, Melbourne, VIC 3800, Australia;

^3^School of Public Health and Preventive Medicine, Monash University, Melbourne, VIC 3004, Australia;

^4^Monash Data Futures Institute, Monash University, Melbourne, VIC 3800, Australia

*To whom correspondence should be addressed.

Corresponding authors. Dong-Jun Yu, School of Computer Science and Engineering, Nanjing University of Science and Technology, China. Email: njyudj@njust.edu.cn; Jiangning Song, Biomedicine Discovery Institute and Department of Biochemistry and Molecular Biology, Monash University, Melbourne, VIC 3800, Australia. Email: jiangning.song@monash.edu.


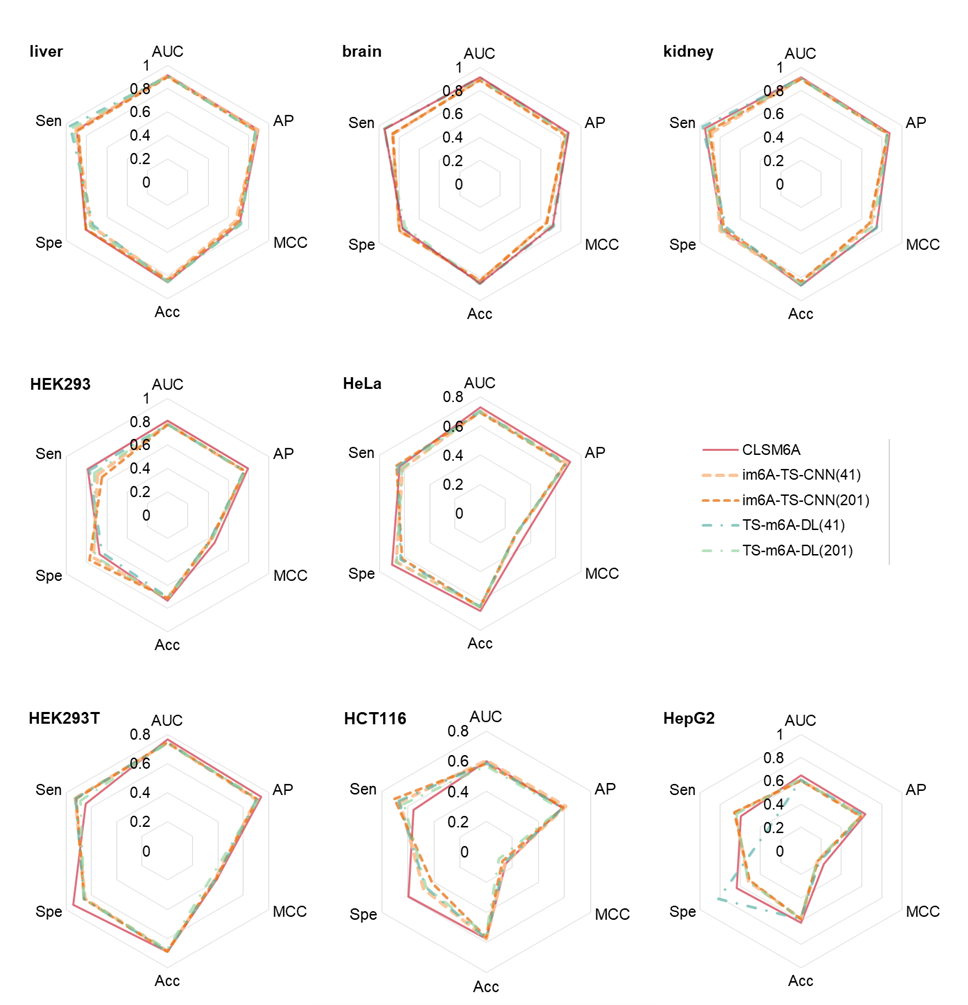


# **Supplementary Figure S1.** Prediction performance on the testing sets in eight cell lines.


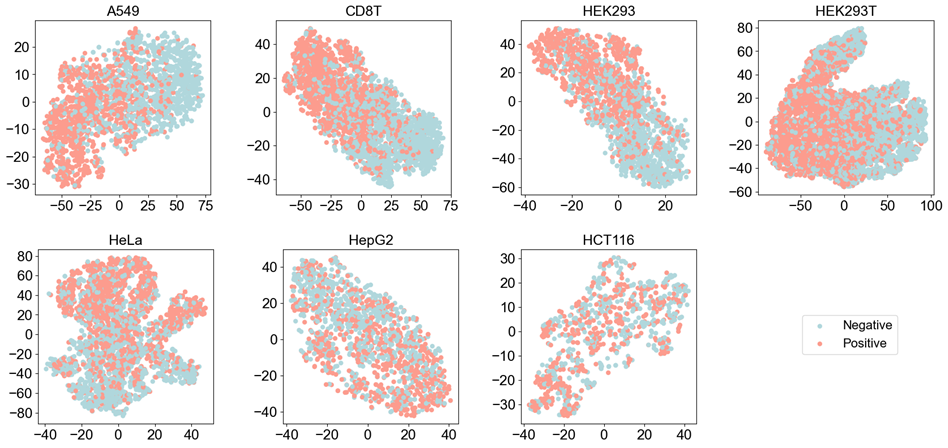


# **Supplementary Figure S2.** The feature space distribution in seven cell lines/tissues.


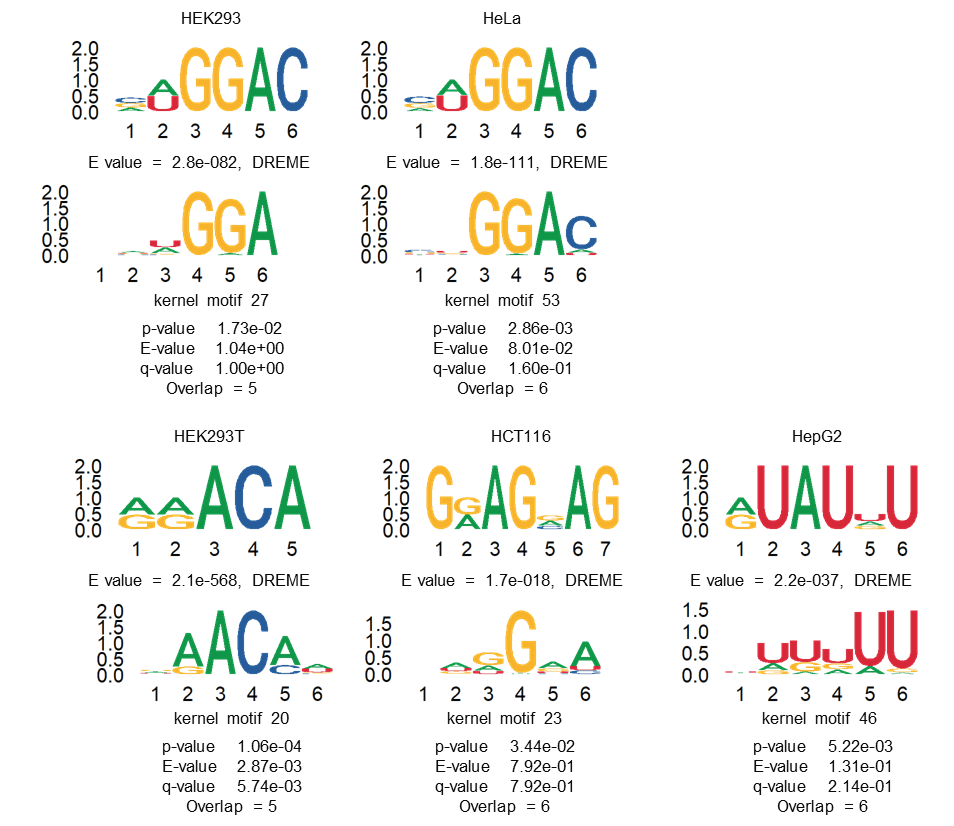


# **Supplementary Figure S3.** Motifs identified from conventional motif finding method DREME can be detected by the first convolutional layer of CLSM6A.


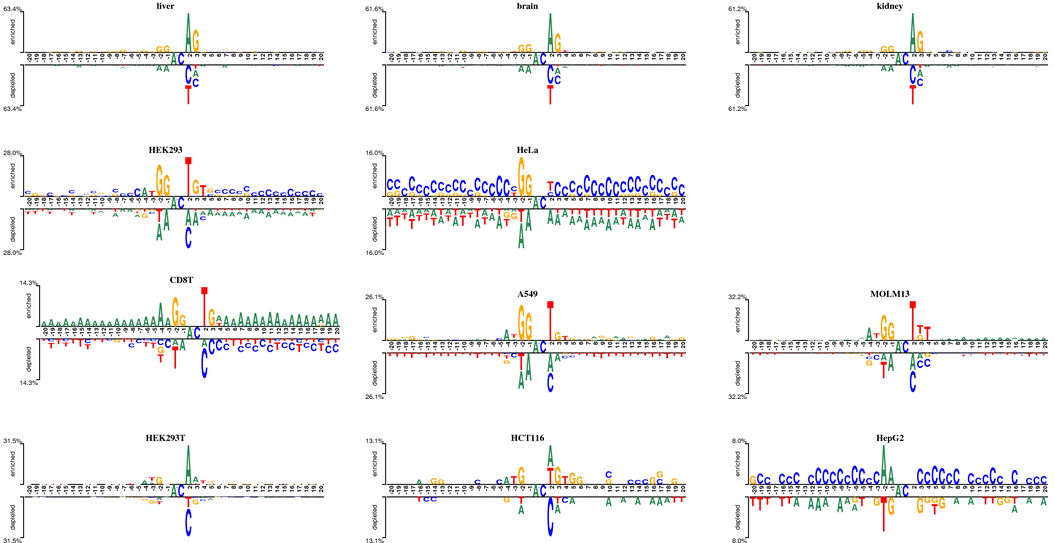


# **Supplementary Figure S4.** The nucleotide distribution surrounding m6A and non-m6A sites by Two Sample Logos.


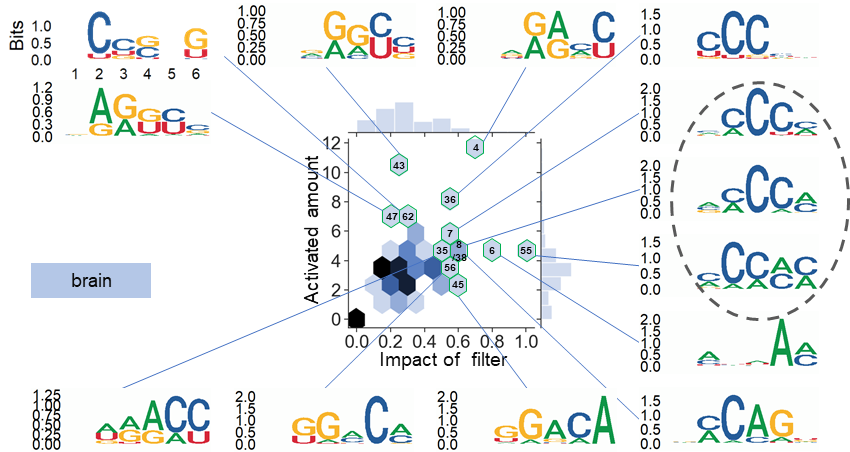


# **Supplementary Figure S5.** Exhibition of the distribution of motifs in brain cell. Motifs with high impact/large activate subsequence amount are displayed.


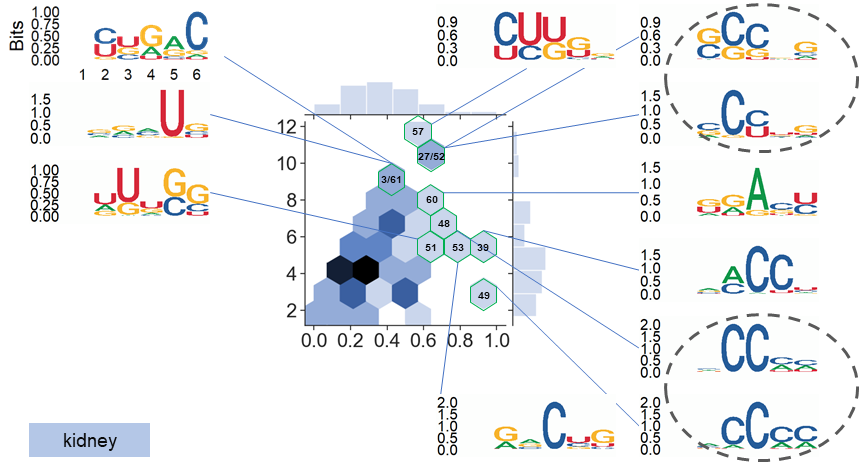


# **Supplementary Figure S6.** Exhibition of the distribution of motifs in kidney cell. Motifs with high impact/large activate subsequence amount are displayed.


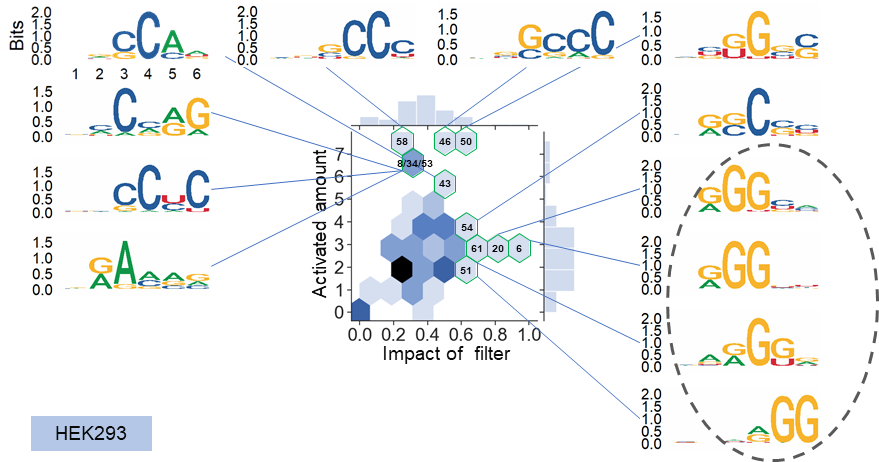


# **Supplementary Figure S7.** Exhibition of the distribution of motifs in HEK293 cell. Motifs with high impact/large activate subsequence amount are displayed.


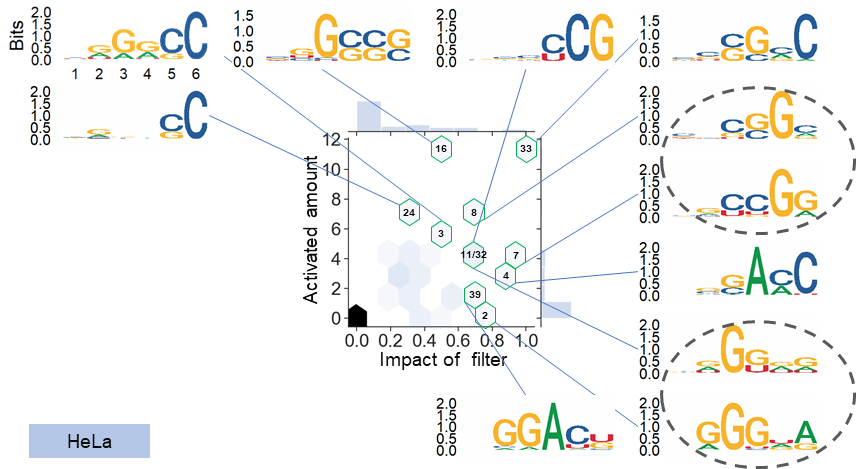


# **Supplementary Figure S8.** Exhibition of the distribution of motifs in HeLa cell. Motifs with high impact/large activate subsequence amount are displayed.


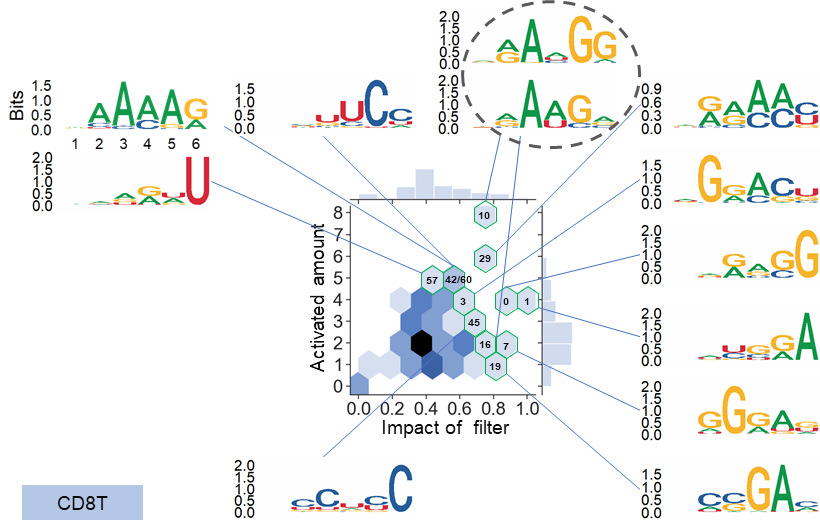


# **Supplementary Figure S9.** Exhibition of the distribution of motifs in CD8T cell. Motifs with high impact/large activate subsequence amount are displayed.


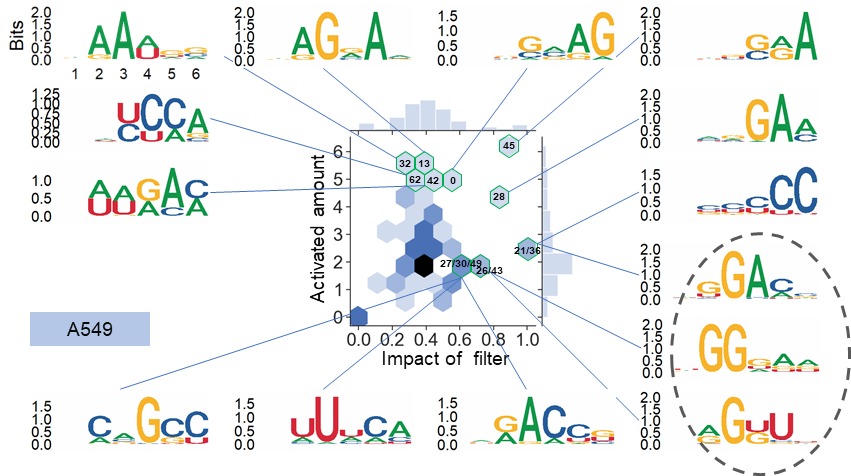


# **Supplementary Figure S10.** Exhibition of the distribution of motifs in A549 cell. Motifs with high impact/large activate subsequence amount are displayed.


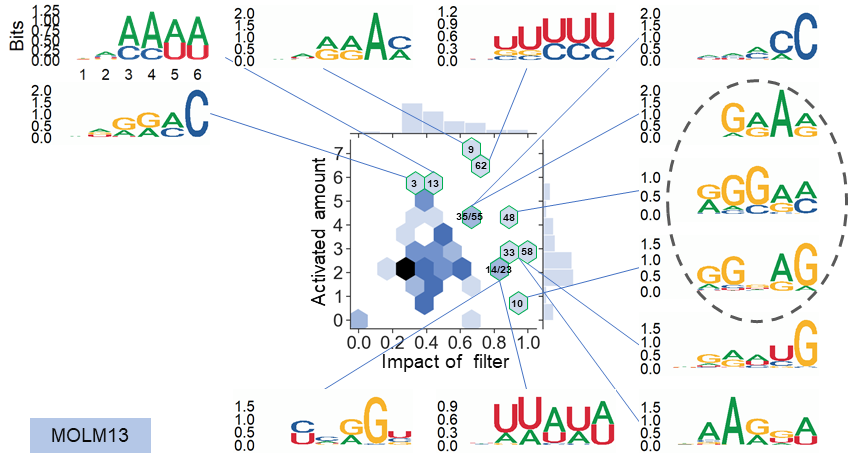


# **Supplementary Figure S11.** Exhibition of the distribution of motifs in MOLM13 cell. Motifs with high impact/large activate subsequence amount are displayed.


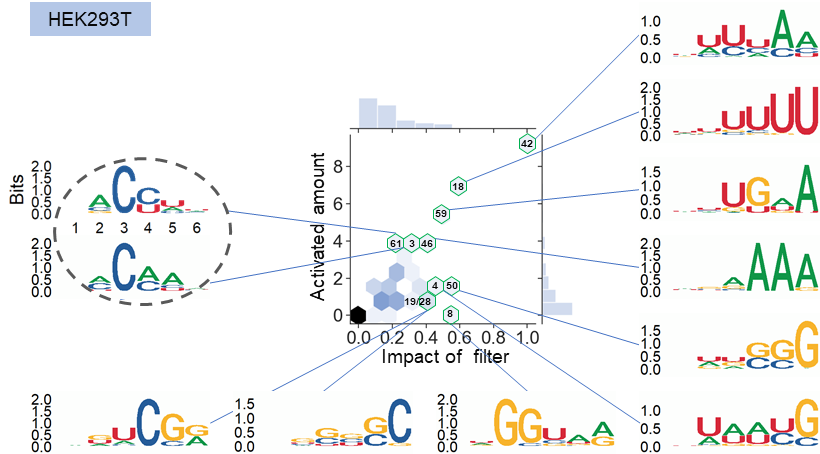


# **Supplementary Figure S12.** Exhibition of the distribution of motifs in HEK293T cell. Motifs with high impact/large activate subsequence amount are displayed.


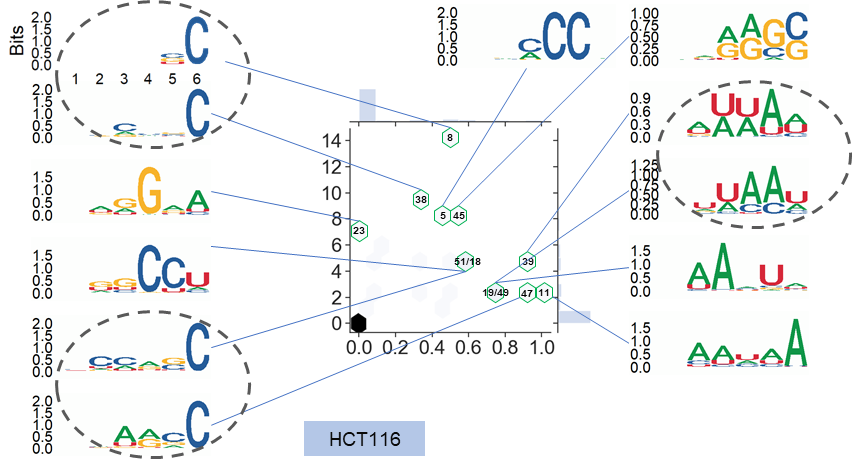


# **Supplementary Figure S13.** Exhibition of the distribution of motifs in HCT116 cell. Motifs with high impact/large activate subsequence amount are displayed.


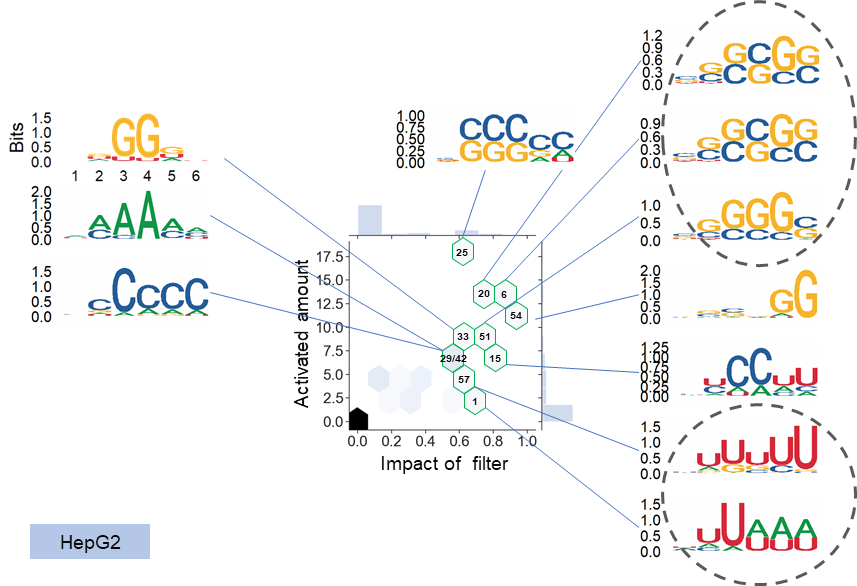


# **Supplementary Figure S14.** Exhibition of the distribution of motifs in HepG2 cell. Motifs with high impact/large activate subsequence amount are displayed.


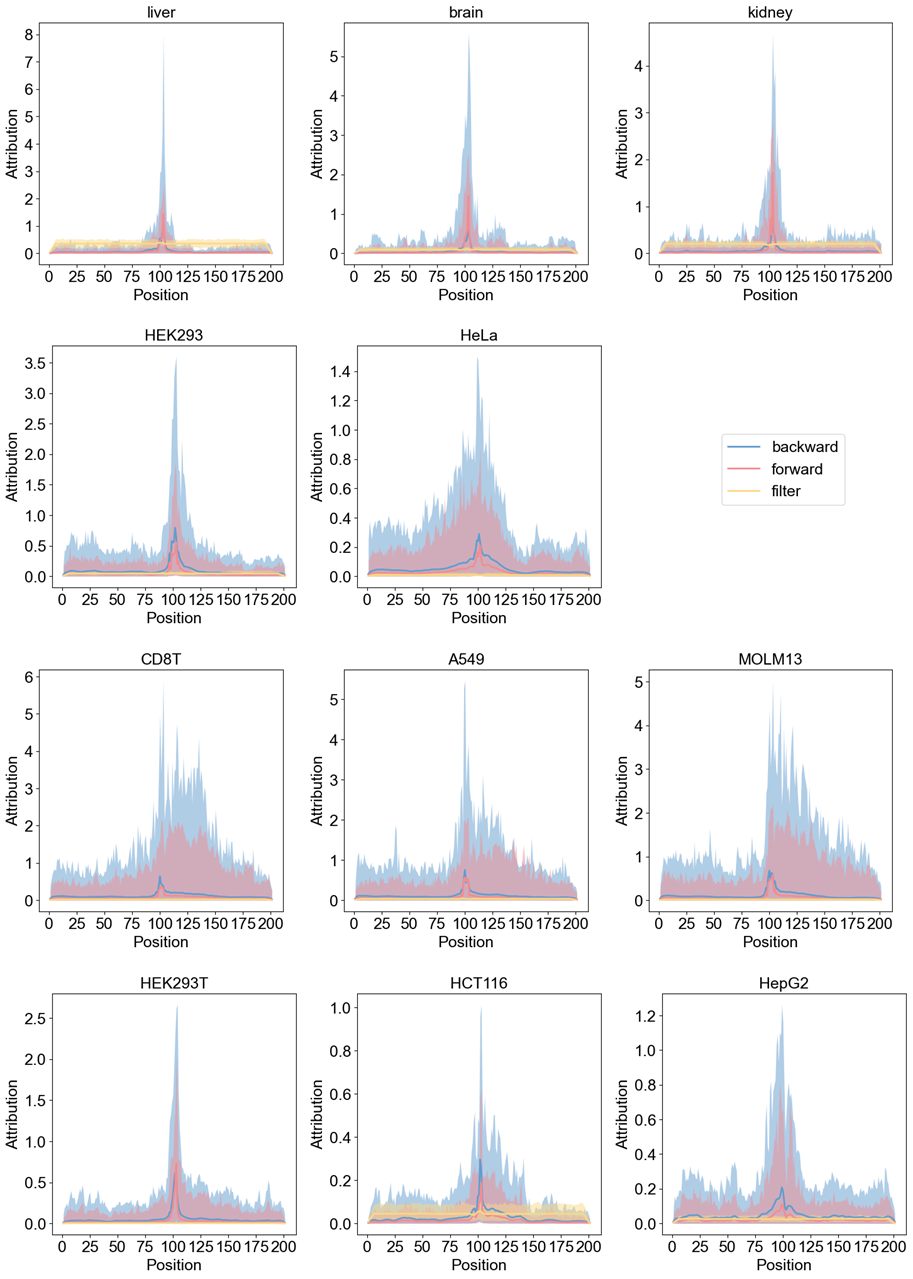


# **Supplementary Figure S15.** Global exhibition of positions the model focuses on via three strategies.


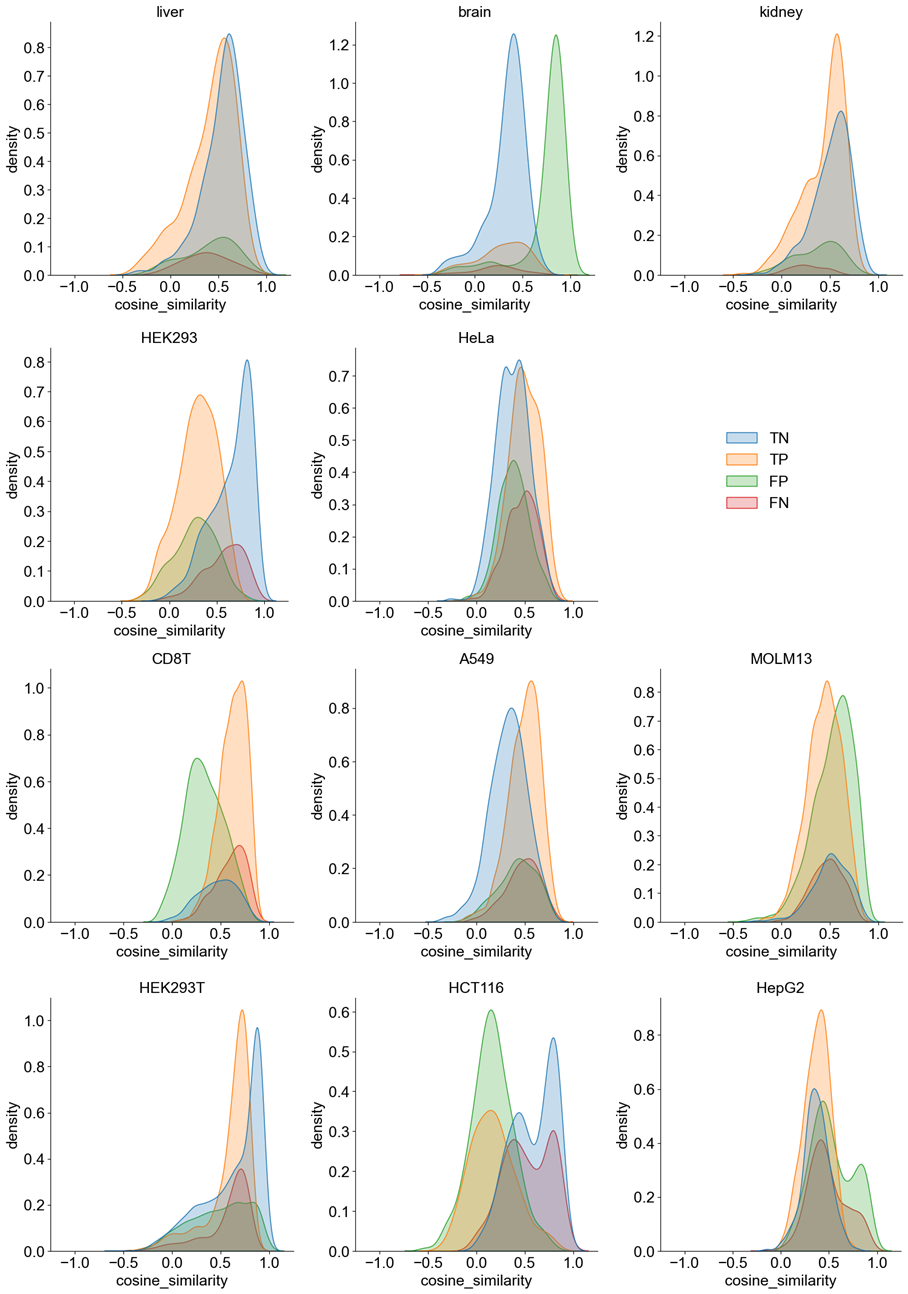


# **Supplementary Figure S16.** Local (single input example) attribution similarity by the two propagation-based interpretation methods.

# **Supplementary Table S1.** Summary of existing approaches for predicting m6A RNA in *H. sapiens*.

| Method/Tool | Year | Algorithm | Features | Evaluation strategy | Species | Tissue/cell line specific | Code or web server |
| --- | --- | --- | --- | --- | --- | --- | --- |
| SRAMP [1] | 2016 | RF | One-hot, KNN,  spectrum | 5-fold cross-validation | H. sapiens  M. musculus | HEK293 cell, CD8+ T cell and A549 cell for H. sapiens  brain and liver for M. musculus | <http://www.cuilab.cn/sramp/> |
| MethyRNA [2] | 2016 | SVM | Chemical property, Nucleotide frequency | Jackknife test | H. sapiens  M. musculus | No | <http://lin-group.cn/server/methyrna/> |
| iRNA-PseCol [3] | 2017 | SVM | Chemical property, Nucleotide frequency | Jackknife test | H. sapiens | No | <http://lin-group.cn/server/iRNA-PseColl/> |
| RAM-NPPS [4] | 2017 | SVM | NPPS | Jackknife test | H. sapiens  S. cerevisiae  A. thaliana | No | <https://server.wei-group.net/RAM-NPPS/> |
| Gene2Vec [5] | 2018 | CNN | One-hot, neighboring methylation state, RNA word, Gene2vec | Training and  validation | H. sapiens  M. musculus | No | <http://server.malab.cn/Gene2vec/> |
| BERMP [6] | 2018 | RF, BGRU | ENAC  word embedding | 5-fold cross-validation | H. sapiens  S. cerevisiae  A. thaliana  M. musculus | No | <http://www.bioinfogo.org/bermp/>  (unavailable now) |
| M6AMRFS [7] | 2018 | XGBoost | Dinucleotide binary, Local position-specific dinucleotide frequency | 10-fold cross-validation, jackknife test | H. sapiens  S. cerevisiae  A. thaliana  M. musculus | No | <http://server.malab.cn/M6AMRFS/>  (unavailable now) |
| HMpre [8] | 2018 | XGBoost | Site location, entropy information, SNP features, binary features, CPD, K-mer | Independent test | H. sapiens | No | No |
| DeepM6ASeq [9] | 2018 | CNN, LSTM | One-hot | Independent test | zebrafish, human, mouse | No | https://github.com/  rreybeyb/DeepM6ASeq/ |
| WHISTLE [10] | 2019 | SVM | NCP, CNF, Genome-derived features | Independent test | H. sapiens | No | <http://whistle-epitranscriptome.com/> |
| DeepPromise [11] | 2019 | CNN | ENAC | Independent test | H. sapiens  M. musculus | No | <http://DeepPromise.erc.monash.edu/> |
| im6A-TS-CNN [12] | 2020 | CNN | One-Hot | 5-fold cross-validation, independent test | human, mouse, and rat | brain, kidney and liver for human  brain, heart, kidney, liver, and testis for mouse  brain, kidney, liver for rat | <https://github.com/liukeweiaway/DeepM6A_cnn/> |
| iRNA-m6A [13] | 2020 | SVM | Physical-chemical property, Mono-nucleotide binary encoding, Nucleotide chemical property | Independent test | human, mouse, and rat | brain, kidney and liver for human  brain, heart, kidney, liver, and testis for mouse  brain, kidney, liver for rat | <http://lin-group.cn/server/iRNA-m6A/service.html/> |
| TS-m6A-DL [14] | 2021 | CNN | One-Hot | 5-fold cross-validation, independent test | human, mouse, and rat | brain, kidney and liver for human  brain, heart, kidney, liver, and testis for mouse  brain, kidney, liver for rat | <https://nsclbio.jbnu.ac.kr/tools/TS-m6A-DL/>  (unavailable now) |
| MultiRM [15] | 2021 | CNN, LSTM | One-hot, HMM, word2vec | Independent test | H. sapiens | No | <https://github.com/Tsedao/MultiRM/> |
| MASS [16] | 2021 | CNN, LSTM, multi-head attention | One-hot | Independent test | zebrafish, human, mouse, rhesus, rat, chimpanzee, pig | No | <https://github.com/mlcb-thu/MASS> |

# **Supplementary Table S2.** The search space of the hyperparameters of CLSM6A.

| Parameters | Search space |
| --- | --- |
| Flanking sequence length | {20, 40, 60, 80, 100} |
| Conv layers | {2, 3, 4} |
| Number of filters | {8, 16, 32, 64} |
| Pooling window size | 2 |
| Dense layer size | {64, 128, 256} |
| Dropout expectation | {0.2, 0.4, 0.6, 0.8} |
| Learning rate | [1e-3, 1e-2] |

# **Supplementary Table S3.** Summary of the models’ hyperparameters.

| Cell line | Conv layers  (filter, filter_length, stride, padding, Dropout) | FC layer  (hidden node, Dropout) | Learning rate | Epoch |
| --- | --- | --- | --- | --- |
| A549 | (64, 5, 1, 2, 0.2)  (64, 5, 1, 2, 0.2)  (64, 5, 1, 2, 0.2) | (256, 0.2) | 1e-3 | 40 |
| brain | (64, 5, 1, 2, 0.2)  (64, 5, 1, 2, 0.2)  (64, 5, 1, 2, 0.2) | (256, 0.2) | 1e-3 | 30 |
| CD8T | (64, 5, 1, 2, 0.2)  (64, 5, 1, 2, 0.2)  (64, 5, 1, 2, 0.2) | (256, 0.2) | 1e-3 | 40 |
| HCT116 | (64, 5, 1, 2, 0.2)  (64, 5, 1, 2, 0.2)  (64, 5, 1, 2, 0.2) | (64, 0.2) | 1e-3 | 30 |
| HEK293 | (64, 5, 1, 2, 0.2)  (64, 5, 1, 2, 0.2)  (64, 5, 1, 2, 0.2) | (256, 0.2) | 1e-3 | 40 |
| HEK293T | (64, 5, 1, 2, 0.2)  (64, 5, 1, 2, 0.2) | (128, 0.2) | 1e-3 | 30 |
| HeLa | (64, 5, 1, 2, 0.2)  (128, 5, 1, 2, 0.2)  (64, 5, 1, 2, 0.2) | (256, 0.2) | 1e-3 | 30 |
| HepG2 | (64, 5, 1, 2, 0.2)  (64, 5, 1, 2, 0.2) | (256, 0.2) | 1e-3 | 30 |
| kidney | (64, 5, 1, 2, 0.2)  (8, 5, 1, 2, 0.2) | (128, 0.2) | 1e-3 | 30 |
| liver | (64, 5, 1, 2, 0.2)  (64, 5, 1, 2, 0.2)  (64, 5, 1, 2, 0.2) | (256, 0.2) | 1e-3 | 30 |
| MOLM13 | (64, 5, 1, 2, 0.2)  (64, 5, 1, 2, 0.2)  (64, 5, 1, 2, 0.2) | (256, 0.2) | 1e-3 | 40 |

# **Supplementary Table S4**. Performances comparison of CLSM6A and the state-of-the-art methods on the independent test datasets.

| Cell line | Method | Acc | AP | AUC | MCC | Sen | Spe |
| --- | --- | --- | --- | --- | --- | --- | --- |
| liver | CLSM6A | 0.8544 | 0.8926 | 0.9167 | 0.7099 | 0.8957 | 0.8104 |
|  | im6A-TS-CNN(41) | 0.8322 | 0.8881 | 0.9074 | 0.6698 | 0.9079 | 0.7516 |
|  | im6A-TS-CNN(201) | 0.8449 | 0.8747 | 0.9010 | 0.6905 | 0.8834 | 0.8039 |
|  | TS-m6A-DL(41) | 0.8544 | 0.8655 | 0.9036 | 0.7237 | 0.9631 | 0.7385 |
|  | TS-m6A-DL(201) | 0.8544 | 0.8684 | 0.9042 | 0.7169 | 0.9386 | 0.7647 |
| brain | CLSM6A | 0.8547 | 0.8767 | 0.9115 | 0.7197 | 0.9422 | 0.7648 |
|  | im6A-TS-CNN(41) | 0.8292 | 0.8593 | 0.8987 | 0.6593 | 0.8592 | 0.7984 |
|  | im6A-TS-CNN(201) | 0.8318 | 0.8363 | 0.8859 | 0.6645 | 0.8643 | 0.7984 |
|  | TS-m6A-DL(41) | 0.8573 | 0.8688 | 0.9101 | 0.7262 | 0.9497 | 0.7622 |
|  | TS-m6A-DL(201) | 0.8471 | 0.8598 | 0.8974 | 0.7082 | 0.9497 | 0.7416 |
| kidney | CLSM6A | 0.8707 | 0.8740 | 0.9125 | 0.7484 | 0.9525 | 0.7801 |
|  | im6A-TS-CNN(41) | 0.8458 | 0.8546 | 0.9041 | 0.6913 | 0.8854 | 0.8018 |
|  | im6A-TS-CNN(201) | 0.8370 | 0.8567 | 0.8984 | 0.6765 | 0.9050 | 0.7616 |
|  | TS-m6A-DL(41) | 0.8663 | 0.8551 | 0.9041 | 0.7478 | 0.9776 | 0.7430 |
|  | TS-m6A-DL(201) | 0.8575 | 0.8589 | 0.9011 | 0.7188 | 0.9273 | 0.7801 |
| HEK293 | CLSM6A | 0.7306 | 0.7952 | 0.8067 | 0.4651 | 0.7912 | 0.6707 |
|  | im6A-TS-CNN(41) | 0.7112 | 0.7570 | 0.7777 | 0.4227 | 0.6898 | 0.7324 |
|  | im6A-TS-CNN(201) | 0.7112 | 0.7599 | 0.7770 | 0.4251 | 0.6507 | 0.7711 |
|  | TS-m6A-DL(41) | 0.7063 | 0.7537 | 0.7754 | 0.4163 | 0.7680 | 0.6452 |
|  | TS-m6A-DL(201) | 0.7179 | 0.7587 | 0.7778 | 0.4359 | 0.7216 | 0.7142 |
| HeLa | CLSM6A | 0.6705 | 0.7144 | 0.7271 | 0.3413 | 0.6397 | 0.7009 |
|  | im6A-TS-CNN(41) | 0.6425 | 0.6868 | 0.6998 | 0.2852 | 0.6193 | 0.6655 |
|  | im6A-TS-CNN(201) | 0.6418 | 0.6731 | 0.6955 | 0.2842 | 0.6622 | 0.6217 |
|  | TS-m6A-DL(41) | 0.6408 | 0.6847 | 0.7020 | 0.2818 | 0.6509 | 0.6308 |
|  | TS-m6A-DL(201) | 0.6425 | 0.6796 | 0.6967 | 0.2852 | 0.6186 | 0.6662 |
| CD8T | CLSM6A | 0.7636 | 0.8151 | 0.8431 | 0.5287 | 0.7892 | 0.7393 |
|  | im6A-TS-CNN(41) | 0.6709 | 0.6964 | 0.7242 | 0.3427 | 0.6879 | 0.6548 |
|  | im6A-TS-CNN(201) | 0.7070 | 0.7567 | 0.7750 | 0.4156 | 0.7352 | 0.6800 |
|  | TS-m6A-DL(41) | 0.6479 | 0.6825 | 0.7113 | 0.3075 | 0.7568 | 0.5442 |
|  | TS-m6A-DL(201) | 0.6928 | 0.7269 | 0.7562 | 0.3858 | 0.6979 | 0.6879 |
| A549 | CLSM6A | 0.7641 | 0.8174 | 0.8451 | 0.5284 | 0.7462 | 0.7817 |
|  | im6A-TS-CNN(41) | 0.7053 | 0.7157 | 0.7668 | 0.4115 | 0.7289 | 0.6822 |
|  | im6A-TS-CNN(201) | 0.7192 | 0.7565 | 0.7898 | 0.4398 | 0.7516 | 0.6875 |
|  | TS-m6A-DL(41) | 0.7240 | 0.7149 | 0.7619 | 0.4524 | 0.7861 | 0.6631 |
|  | TS-m6A-DL(201) | 0.6582 | 0.6902 | 0.7226 | 0.3240 | 0.7537 | 0.5646 |
| MOLM13 | CLSM6A | 0.7893 | 0.8464 | 0.8674 | 0.5789 | 0.7993 | 0.7795 |
|  | im6A-TS-CNN(41) | 0.7228 | 0.7775 | 0.7926 | 0.4457 | 0.7115 | 0.7340 |
|  | im6A-TS-CNN(201) | 0.7477 | 0.7994 | 0.8247 | 0.4955 | 0.7432 | 0.7522 |
|  | TS-m6A-DL(41) | 0.7281 | 0.7774 | 0.7918 | 0.4586 | 0.6699 | 0.7854 |
|  | TS-m6A-DL(201) | 0.7310 | 0.7865 | 0.8057 | 0.4625 | 0.7029 | 0.7587 |
| HEK293T | CLSM6A | 0.6926 | 0.7412 | 0.7654 | 0.3879 | 0.6442 | 0.7420 |
|  | im6A-TS-CNN(41) | 0.6884 | 0.7012 | 0.7420 | 0.3773 | 0.7262 | 0.6498 |
|  | im6A-TS-CNN(201) | 0.6893 | 0.7093 | 0.7439 | 0.3787 | 0.7171 | 0.6609 |
|  | TS-m6A-DL(41) | 0.6902 | 0.7048 | 0.7430 | 0.3807 | 0.7227 | 0.6571 |
|  | TS-m6A-DL(201) | 0.6744 | 0.7187 | 0.7390 | 0.3487 | 0.6885 | 0.6600 |
| HCT116 | CLSM6A | 0.5754 | 0.5835 | 0.6025 | 0.1519 | 0.5584 | 0.5934 |
|  | im6A-TS-CNN(41) | 0.5742 | 0.6072 | 0.6063 | 0.1458 | 0.6610 | 0.4823 |
|  | im6A-TS-CNN(201) | 0.5631 | 0.5903 | 0.5835 | 0.1235 | 0.7040 | 0.4141 |
|  | TS-m6A-DL(41) | 0.5717 | 0.5833 | 0.5888 | 0.1408 | 0.6706 | 0.4671 |
|  | TS-m6A-DL(201) | 0.5496 | 0.5745 | 0.5710 | 0.0959 | 0.6324 | 0.4621 |
| HepG2 | CLSM6A | 0.6135 | 0.6353 | 0.6465 | 0.2267 | 0.5933 | 0.6331 |
|  | im6A-TS-CNN(41) | 0.5817 | 0.6045 | 0.6089 | 0.1668 | 0.6494 | 0.5160 |
|  | im6A-TS-CNN(201) | 0.5810 | 0.5955 | 0.6050 | 0.1661 | 0.6580 | 0.5062 |
|  | TS-m6A-DL(41) | 0.5817 | 0.6185 | 0.6115 | 0.1788 | 0.3362 | 0.8200 |
|  | TS-m6A-DL(201) | 0.5789 | 0.5895 | 0.6170 | 0.1606 | 0.6393 | 0.5202 |
| Average | CLSM6A | **0.7436** | **0.7811** | **0.8040** | **0.4897** | **0.7593** | **0.7269** |
|  | im6A-TS-CNN(41) | 0.7095 | 0.7408 | 0.7662 | 0.4198 | 0.7388 | 0.6790 |
|  | im6A-TS-CNN(201) | 0.7158 | 0.7462 | 0.7709 | 0.4327 | 0.7522 | 0.6780 |
|  | TS-m6A-DL(41) | 0.7153 | 0.7372 | 0.7640 | 0.4377 | 0.7501 | 0.6779 |
|  | TS-m6A-DL(201) | 0.7095 | 0.7374 | 0.7626 | 0.4220 | 0.7519 | 0.6655 |

# **Supplementary Text S1**

For each cell line/tissue, 10% of the samples were randomly divided as the independent test dataset, and the remaining 90% were used as the training dataset. We conducted experiments on training datasets with the flanking sequence length ranging from 20 nt to 100 nt at intervals of 20 nt to determine the appropriate sequence length. Specifically, for each cell line/tissue, the training dataset was randomly divided into five non-overlapping subsets. In each validation step, four subsets were used to train the model on a set sequence length, while the remaining subset was used to test the performance of the trained model. The AUC values and AP values of the 5-fold cross-validation were displayed.

# **Reference**

[1] Zhou Y, Zeng P, Li YH, Zhang Z, Cui Q. SRAMP: prediction of mammalian N6-methyladenosine (m6A) sites based on sequence-derived features. Nucleic acids research 2016;44:e91.

[2] Chen W, Tang H, Lin H. MethyRNA: a web server for identification of N(6)-methyladenosine sites. Journal of biomolecular structure & dynamics 2017;35:683-7.

[3] Feng P, Ding H, Yang H, Chen W, Lin H, Chou KC. iRNA-PseColl: Identifying the Occurrence Sites of Different RNA Modifications by Incorporating Collective Effects of Nucleotides into PseKNC. Molecular therapy Nucleic acids 2017;7:155-63.

[4] Xing P, Su R, Guo F, Wei L. Identifying N(6)-methyladenosine sites using multi-interval nucleotide pair position specificity and support vector machine. Scientific reports 2017;7:46757.

[5] Zou Q, Xing P, Wei L, Liu B. Gene2vec: gene subsequence embedding for prediction of mammalian N (6)-methyladenosine sites from mRNA. RNA (New York, NY) 2019;25:205-18.

[6] Huang Y, He N, Chen Y, Chen Z, Li L. BERMP: a cross-species classifier for predicting m(6)A sites by integrating a deep learning algorithm and a random forest approach. International journal of biological sciences 2018;14:1669-77.

[7] Qiang X, Chen H, Ye X, Su R, Wei L. M6AMRFS: Robust Prediction of N6-Methyladenosine Sites With Sequence-Based Features in Multiple Species. Frontiers in genetics 2018;9:495.

[8] Zhao Z, Peng H, Lan C, Zheng Y, Fang L, Li J. Imbalance learning for the prediction of N(6)-Methylation sites in mRNAs. BMC genomics 2018;19:574.

[9] Zhang Y, Hamada M. DeepM6ASeq: prediction and characterization of m6A-containing sequences using deep learning. BMC bioinformatics 2018;19:524.

[10] Chen K, Wei Z, Zhang Q, Wu X, Rong R, Lu Z, et al. WHISTLE: a high-accuracy map of the human N6-methyladenosine (m6A) epitranscriptome predicted using a machine learning approach. Nucleic acids research 2019;47:e41.

[11] Chen Z, Zhao P, Li F, Wang Y, Smith AI, Webb GI, et al. Comprehensive review and assessment of computational methods for predicting RNA post-transcriptional modification sites from RNA sequences. Briefings in bioinformatics 2020;21:1676-96.

[12] Liu K, Cao L, Du P, Chen W. im6A-TS-CNN: Identifying the N(6)-Methyladenine Site in Multiple Tissues by Using the Convolutional Neural Network. Molecular therapy Nucleic acids 2020;21:1044-9.

[13] Dao FY, Lv H, Yang YH, Zulfiqar H, Gao H, Lin H. Computational identification of N6-methyladenosine sites in multiple tissues of mammals. Computational and structural biotechnology journal 2020;18:1084-91.

[14] Abbas Z, Tayara H, Zou Q, Chong KT. TS-m6A-DL: Tissue-specific identification of N6-methyladenosine sites using a universal deep learning model. Computational and structural biotechnology journal 2021;19:4619-25.

[15] Song Z, Huang D, Song B, Chen K, Song Y, Liu G, et al. Attention-based multi-label neural networks for integrated prediction and interpretation of twelve widely occurring RNA modifications. Nature communications 2021;12:4011.

[16] Xiong Y, He X, Zhao D, Tian T, Hong L, Jiang T, et al. Modeling multi-species RNA modification through multi-task curriculum learning. Nucleic acids research 2021;49:3719-34.
